# Supplementary material for: Comparative analysis of metabolic models of microbial communities reconstructed from automated tools and consensus approaches
Source: NPJ Syst Biol Appl. 2024 May 23;10:54. doi: 10.1038/s41540-024-00384-y (PMC11116368; doi:10.1038/s41540-024-00384-y)
Supplement: Supplementary file 1 — Supplemental material [file 41540_2024_384_MOESM1_ESM.pdf]

Comparative analysis of metabolic models of microbial communities reconstructed from automated tools and consensus approaches

Supplementary Information

Yunli Eric Hsieh,[1,2,3] Kshitij Tandon,[3] Heroen Verbruggen,[3] Zoran Nikoloski[1,2#]

[1]Institute of Biochemistry and Biology, Bioinformatics Department, University of Potsdam, Potsdam, Germany

[2]Systems Biology and Mathematical Modeling Group, Max Planck Institute of Molecular Plant Physiology, Potsdam, Germany

[3]School of BioSciences, The University of Melbourne, Parkville, VIC, Australia

#Address correspondence to Zoran Nikoloski, Nikoloski@mpimp-golm.mpg.de

Supplementary Figures

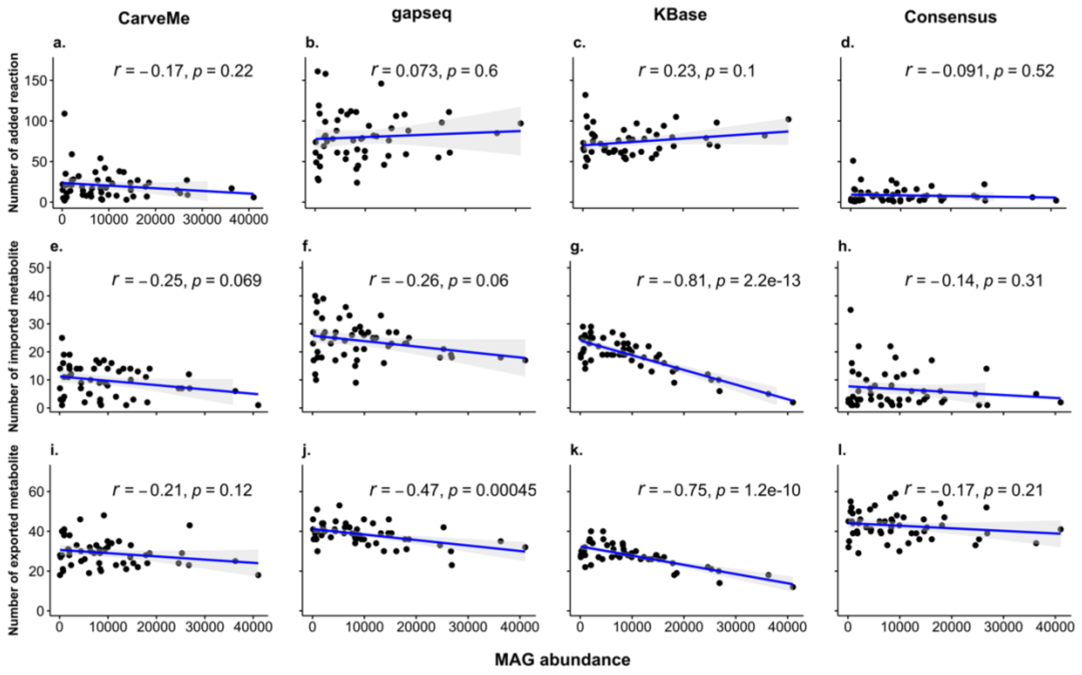

Supplementary Fig 1. Association between MAG abundance and gap-filling results a descending order in different reconstructions of seawater bacteria community model. Pearson correlation coefficient was employed to evaluate the association between MAG abundance and the number of added reactions (a - d), imported metabolites (e - h), and exported metabolites (i - l), for each of the four reconstruction approaches: CarveMe, gapseq, KBase, and the consensus method used in COMMIT. The correlation coefficient (*r*) and corresponding p-value (*p*) were determined.

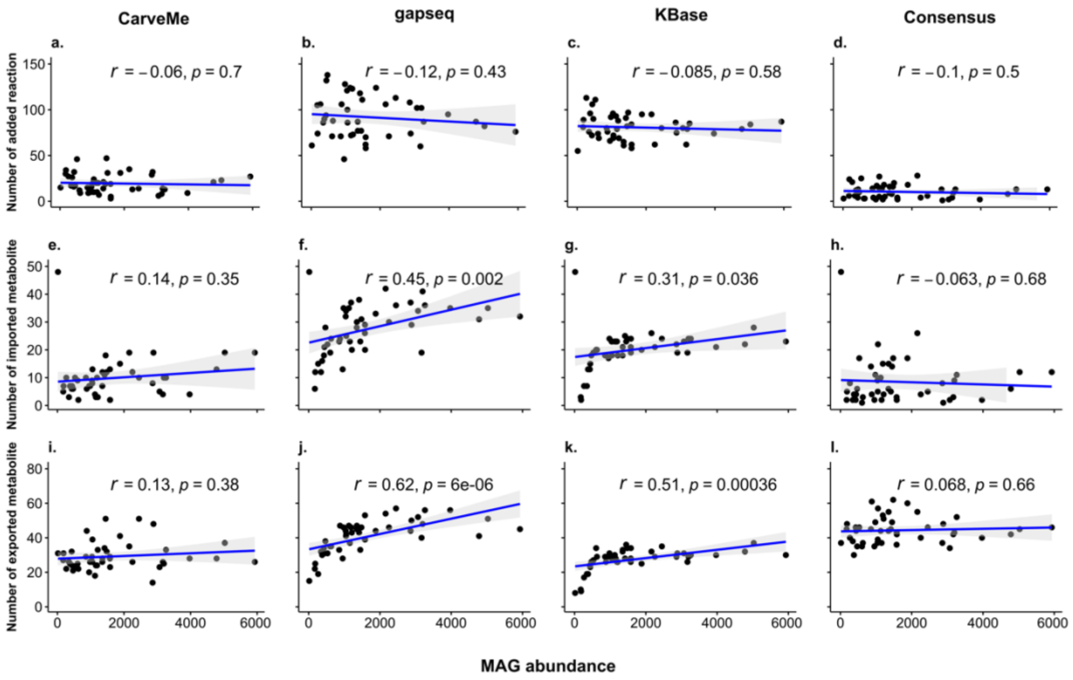

Supplementary Fig 2. Association between MAG abundance and gap-filling results with an ascending order of MAG abundance in different reconstructions of coral-associated bacteria community model. Pearson correlation coefficient was employed to evaluate the association between MAG abundance and the number of added reactions (a - d), imported metabolites (e - h), and exported metabolites (i - l), for each of the four reconstruction approaches: CarveMe, gapseq, KBase, and the consensus method used in COMMIT. The correlation coefficient (*r*) and corresponding p-value (*p*) were determined.

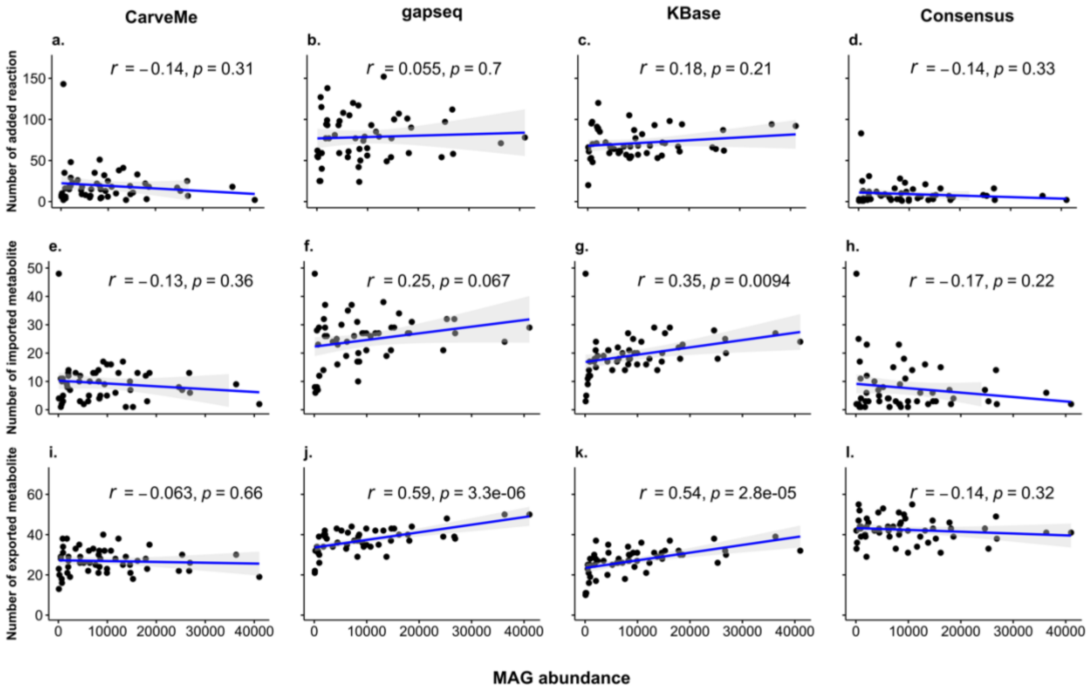

Supplementary Fig 3. Association between MAG abundance and gap-filling results with an ascending order of MAG abundance in different reconstructions of seawater bacteria community model. Pearson correlation coefficient was employed to evaluate the association between MAG abundance and the number of added reactions (a - d), imported metabolites (e - h), and exported metabolites (i - l), for each of the four reconstruction approaches: CarveMe, gapseq, KBase, and the consensus method used in COMMIT. The correlation coefficient (*r*) and corresponding p-value (*p*) were determined.

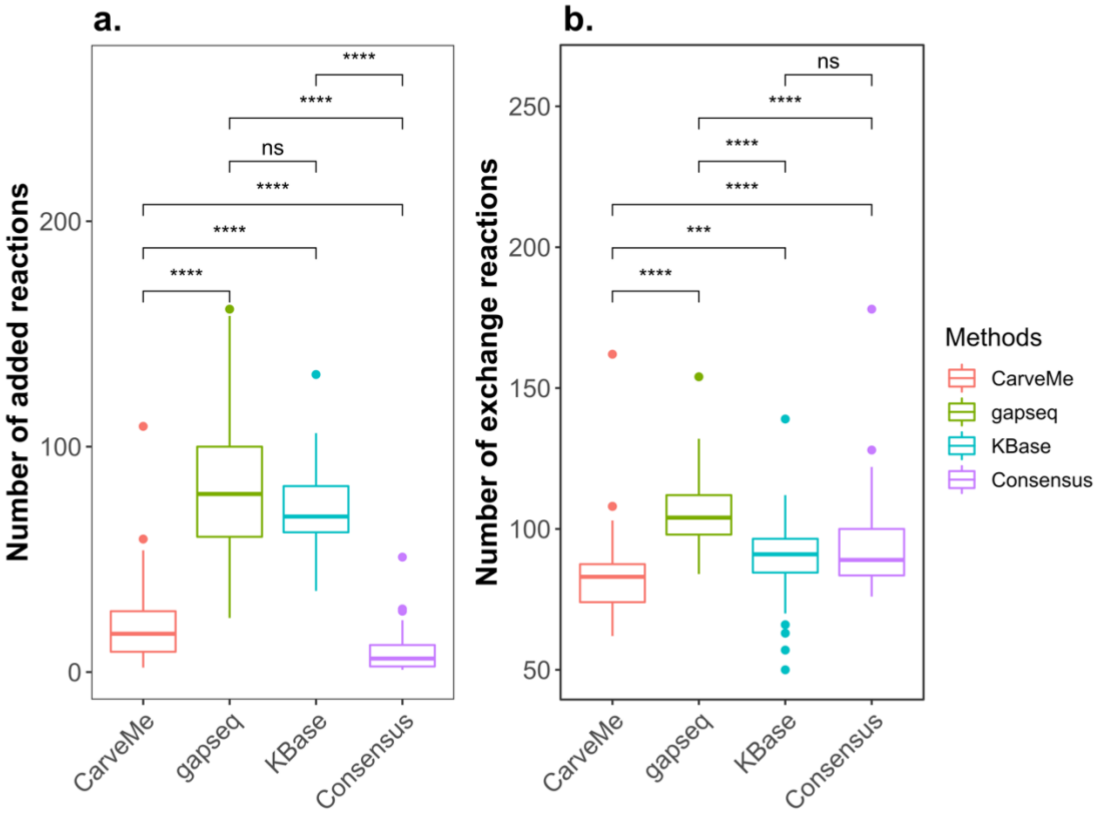

Supplementary Fig 4. Comparison of functional models in different reconstructions of the seawater bacteria community model. The size of gap-filling solutions and the number of exchange reactions in functional models, that can simulate growth, were compared using the Wilcoxon Rank test (* $p < 0.05$; *** $p < 0.001$; **** $p < 0.0001$; ns $p > 0.05$). Panels a and b represent the size of gap-filling solutions and the number of exchange reactions, respectively.

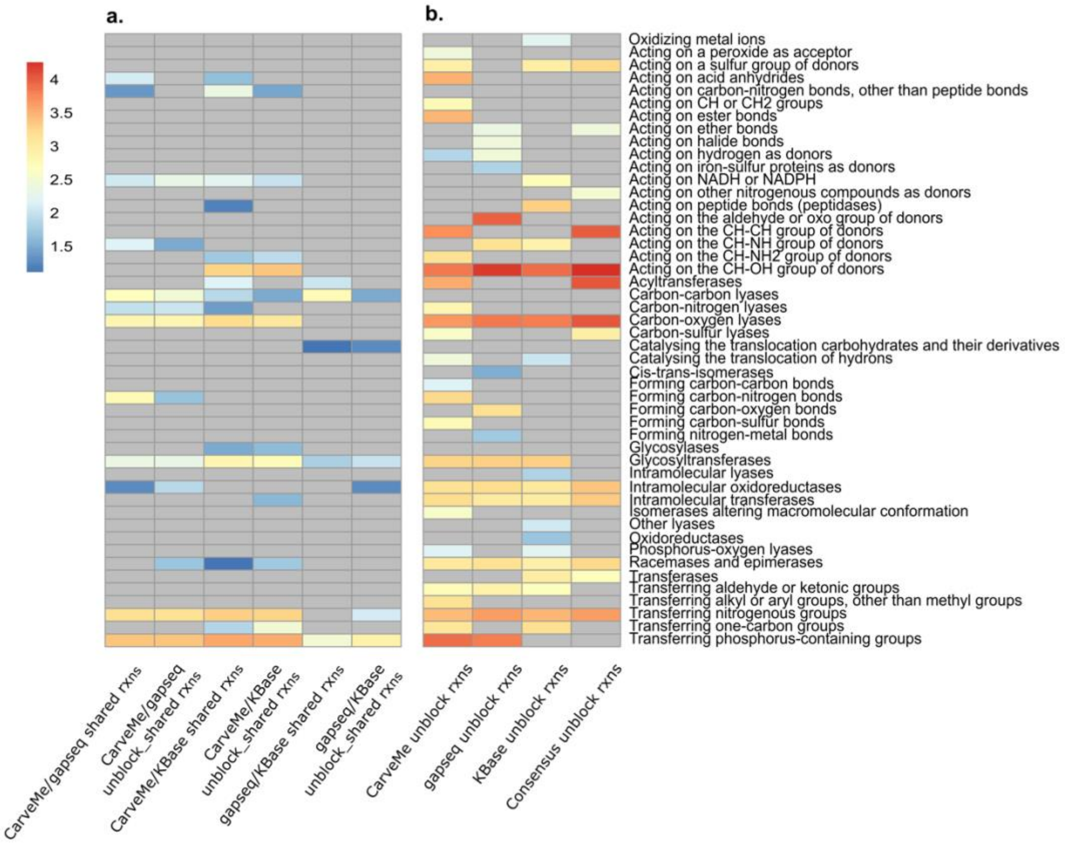

Supplementary Fig 5. Enriched enzyme subclasses in the seawater bacteria community model from different reconstructions. The pairwise comparison of enriched enzyme subclasses in a. shared reactions between each reconstruction and b. in the community models reconstructed by different approaches, analyzed using the hypergeometric test. The abundance of enzyme subclasses is represented in a logarithmic scale and depicted using a color scale ranging from blue to red, with higher numbers indicating greater abundance. Grey color indicates the absence of enriched enzyme subclasses.

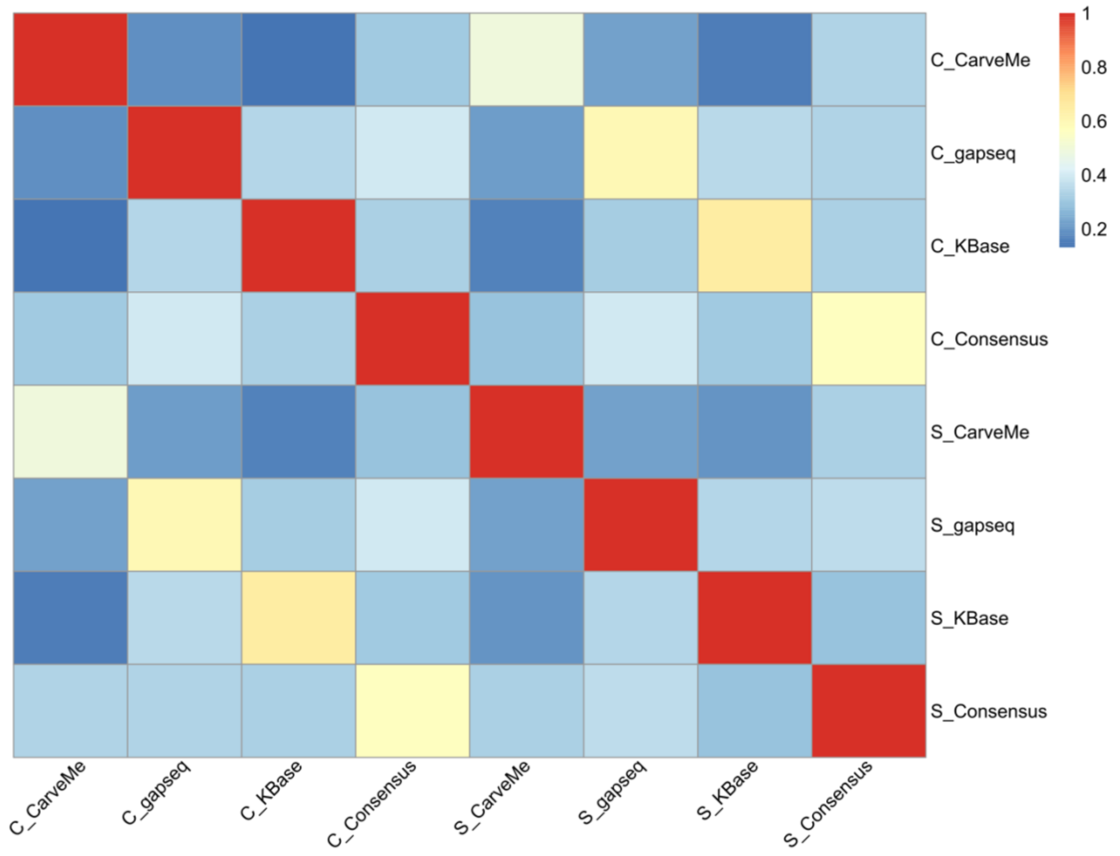

Supplementary Fig 6. Jaccard index matrix of exchanged metabolites between different reconstruction approaches across the coral-associated and seawater bacteria communities. The pairwise comparison of exchanged metabolites within the community, derived from different approaches, was assessed using the Jaccard similarity. The labels 'C_' and 'S_' correspond to the coral-associated bacteria and seawater bacteria communities, respectively. The Jaccard index, ranging from 0 to 1, is visualized using a color scale that transitions from blue to red.

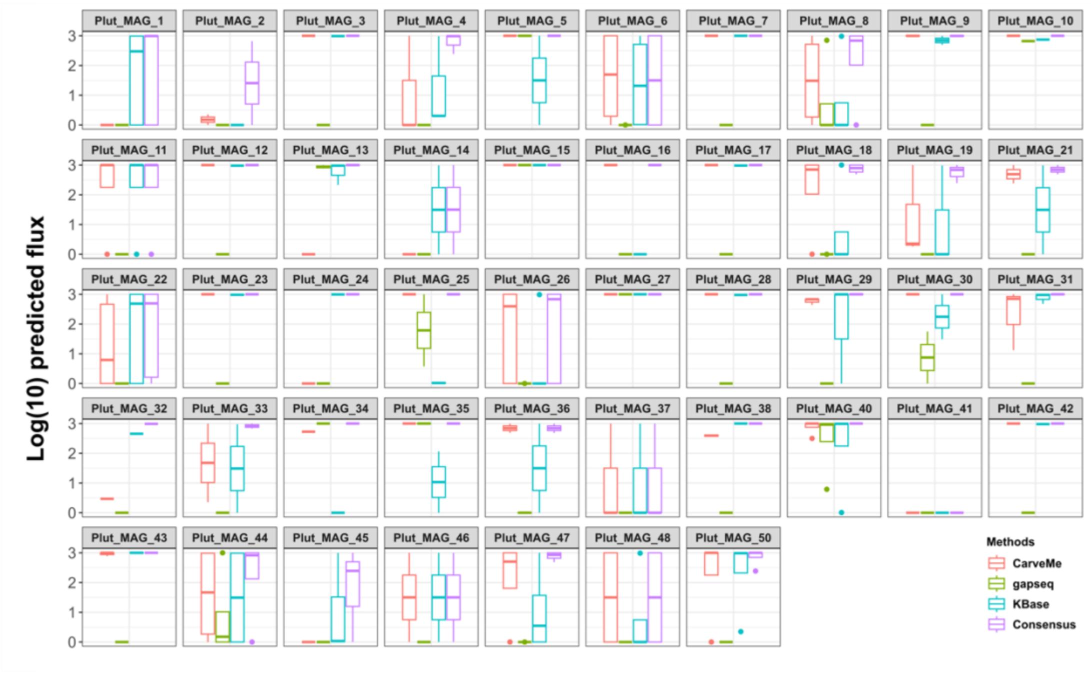

Supplementary Fig 7. Maximum flux comparison of exported metabolites in coral-associated bacteria models reconstructed from the same MAG. The log10 + 1 transformation was applied to depict the maximum predicted flux of the same exported metabolites in the models reconstructed from the same MAG.

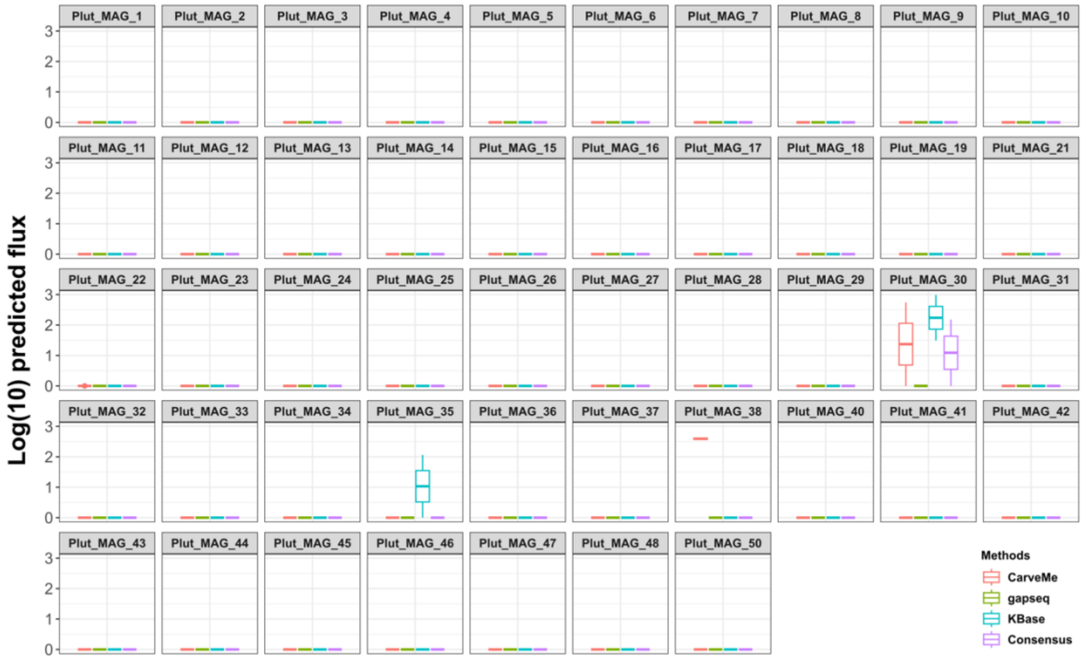

Supplementary Fig 8. Minimum flux comparison of exported metabolites in coral-associated bacteria models reconstructed from the same MAG. The log10 + 1 transformation was applied to depict the minimum predicted flux of the same exported metabolites in the models reconstructed from the same MAG.

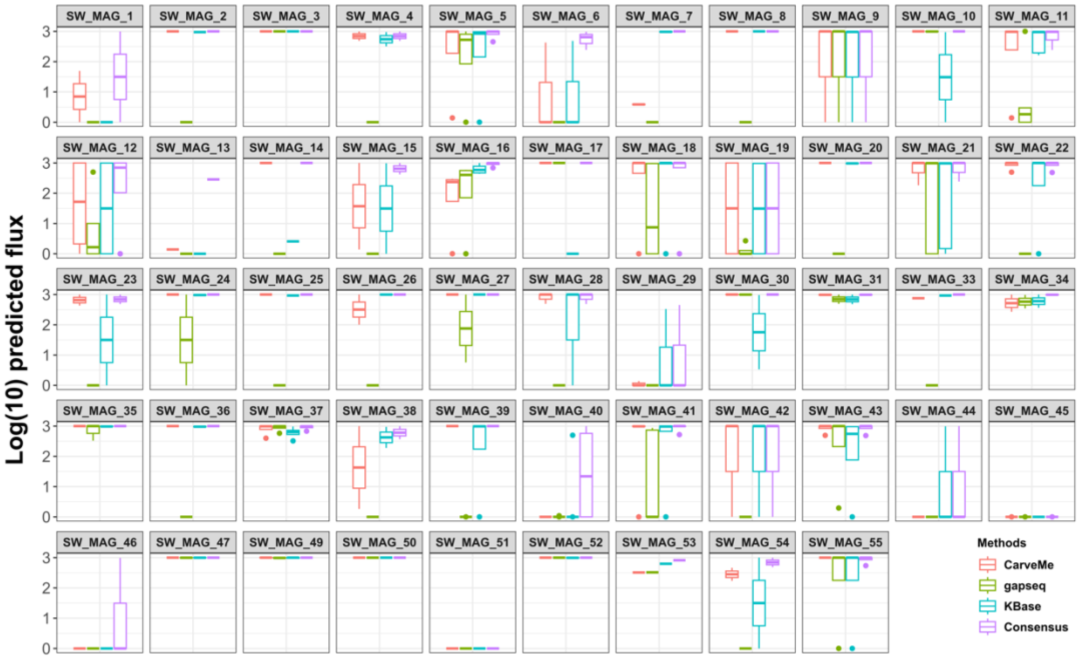

Supplementary Fig 9. Maximum flux comparison of exported metabolites in seawater bacteria models reconstructed from the same MAG. The log10 + 1 transformation was applied to depict the maximum predicted flux of the same exported metabolites in the models reconstructed from the same MAG.

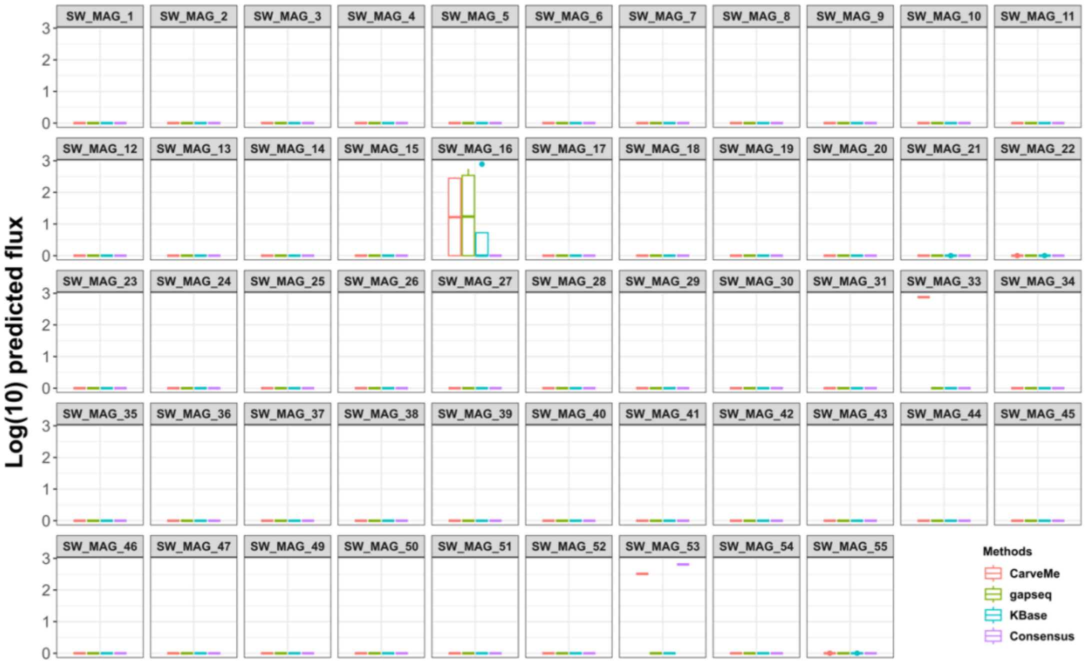

Supplementary Fig 10. Minimum flux comparison of exported metabolites in seawater bacteria models reconstructed from the same MAG. The log10 + 1 transformation was applied to depict the minimum predicted flux of the same exported metabolites in the models reconstructed from the same MAG.

Supplementary Table

Supplementary Table 1. The M9 medium used for gap-filling.

MetaNetXID	Metabolite name
MNXM128	Ca(2+)
MNXM731166	Cu(2+)
MNXM90960	Co(2+)
MNXM95	K(+)
MNXM735978	Cl(-)
MNXM726711	Fe(2+)
MNXM726712	Fe(3+)
MNXM1137670	D-Glucose
WATER	H2O
MNXM1	H(+)
MNXM531925	Mg
MNXM2255	Mn(2+)
MNXM726092	Molybdate
MNXM27	Na(+)
MNXM729302	NH4(+)
MNXM731950	Ni(2+)
MNXM735438	O2
MNXM9	Phosphate

MNXM58	Sulfate
MNXM729215	Zn(2+)
MNXM653	Mg(2+)
